# A new raspberry ketone synthesis gene *RinPKS4* identified in *Rubus idaeus* L. by transcriptome analysis

**DOI:** 10.1371/journal.pone.0306615

**Published:** 2024-08-22

**Authors:** Zhimin He, Xihuan Yan, Junxin Zhang, Kang Hu, Mengzhe Ou, Chaojun Wei, Aizhen Yang, Jing Li, Tiran Huang, Mingfeng Yang, Lanqing Ma

**Affiliations:** 1 College of Bioscience and Resources Environment, Beijing University of Agriculture, Beijing, China; 2 Key Laboratory for Northern Urban Agriculture Ministry of Agriculture and Rural Afairs, Beijing University of Agriculture, Beijing, China; Shandong Agricultural University, CHINA

## Abstract

Raspberry ketone accounts for the characteristic aroma of the raspberry fruit. In order to explore the genes involved in raspberry ketone synthesis, the transcriptome in fruit tissues of two red raspberry varieties “Polka” and “Orange legend”, were sequenced and 24213 single genes were obtained. As the red raspberry fruit ripening, genes involved in flavonoid and anthocyanin synthesis were up-regulated, while those associated with lignin synthesis were down-regulated. A gene (*RinPKS4*) highly related to raspberry ketone synthesis was identified by transcriptome analysis, and *RinPKS4* gene was over-expressed in raspberry in order to further understand the function of *RinPKS4* gene in raspberry ketone synthesis. The results showed that the gene expression level of *RinPKS4* in the leaf tissues of a transgenic lines increased by about 4-fold and the content of raspberry ketone increased by 42.64% compared with the wide type. This study lays a theoretical foundation for further study on the synthesis and regulation of raspberry ketone in red raspberry.

## 1. Introduction

Red raspberry (*Rubus idaeus* L.), known as “golden fruit” [1], is delicious, aromatic and contain many nutritious active ingredients, such as anthocyanin, raspberry ketone, ellagic acid, salicylic acid, vitamin A etc. [2]. Red raspberry can not only be used for fresh food, but also can be used for processing wine, juice, etc. [3]. Therefore, red raspberry has a high economic value and a broad market prospects [4]. Red raspberry fruit has a unique aroma that came from raspberry ketone, a characteristic compound in red raspberry [5].

Raspberry ketone [4-(4-hydroxyphenyl)-butan-2-one] is a low-calorie, warm-scented plant spice with unique flavor [6]. In addition, raspberry ketone has a great potential in anti-cancer, anti-inflammation, prevention of cardio-cerebrovascular and diabetes [7]. Raspberry ketone is mainly found in the fruit of the red raspberry, but yields are typically very low, the content is usually 1~4 mg kg^-1^ raspberry [8]. Up to $20,000 kg^-1^ may be paid for the natural compound [9]. Due to its major values, red raspberry has recently been considered a valuable fruit. The biosynthesis of raspberry ketone begins with the phenylpropanoid pathway (S1 Fig) [10]. P-Hydroxybenzalacetone is the precursor of raspberry ketone, and the Benzalacetone synthase (*BAS*), which catalyzes this reaction, is undoubtedly the key enzyme to regulate the synthesis of raspberry ketone [11]. BAS belongs to the plant type III polyketide synthase (PKS) superfamily and plays an important role in plant growth and development. However, BAS has not been isolated from red raspberry and its function has not been identified.

To investigate the genes involved in raspberry ketone synthesis, we generated transcriptomes of green fruit and ripe fruit of “Polka” and “Orange legend” using the Illumina HiSeq platform. Green fruit indicates the immature stage of the raspberry, and red fruit is the ripe stage of the raspberry. The two raspberry varieties had the most significant difference in raspberry ketones between green and red fruits. Therefore, two representative periods of two varieties of raspberries were selected as samples. The assembled transcripts were functionally annotated. A novel plant type III polyketide synthase (PKS) superfamily gene, named *RinPKS4*, was obtained by analyzing and screening the transcript data, and its function was identified. This study provides a theoretical basis for analyzing the synthesis of raspberry ketone in red raspberry, and lays a foundation for perfecting the biosynthesis pathway of raspberry ketone and improving the quality of red raspberry.

## 2. Materials and methods

### 2.1 Plant material and tissue collection

**Fig 1:**
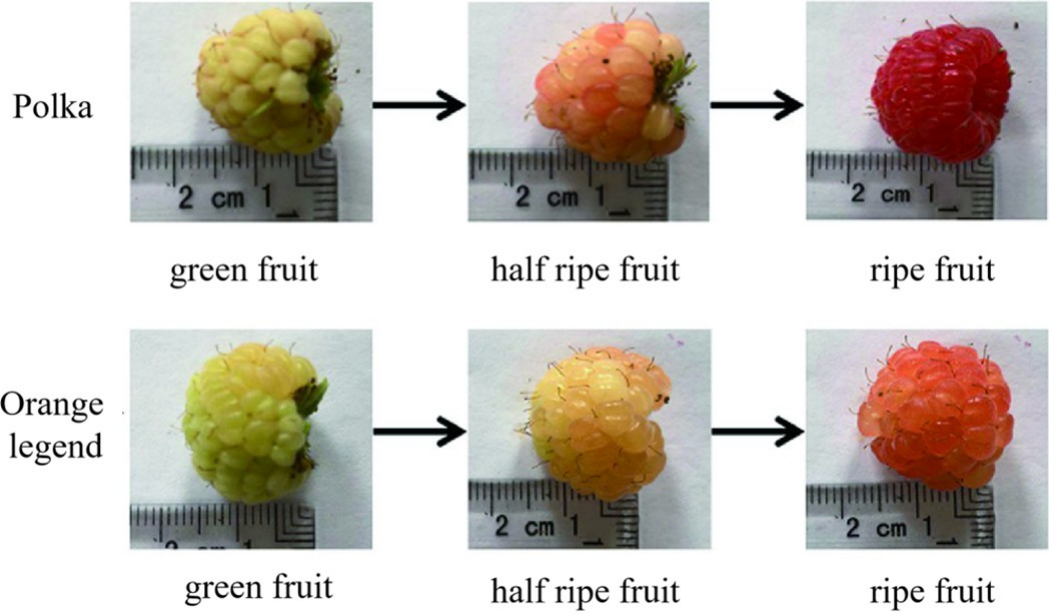
The red raspberry at different development stage [12].

In this study, two different varieties of red raspberry “Polka” and “Orange legend” were maintained in the plant garden and in a greenhouse at the Beijing University of Agriculture, Beijing, China. The fruit sampling is divided into three development stages (Fig 1), the green fruit (15d after full bloom), half ripe fruit (27 d after full bloom), ripe fruit (34 d after full bloom). Fruit samples at the different development stage were collected from at least five plant individuals. All the samples were immediately frozen in liquid nitrogen and stored at -80°C until RNA extraction. At least three fruits were combined to form one biological replicate and each sample has three biological replicates.

### 2.2 Library construction and transcriptome assembly

Total RNA was extracted from the 0.5 g of red raspberry tissue at different development stages using RNA prep pure Plant Kit (Autolabtech, Beijing, China) according to the manufacturer’s instructions. Each sample has three replicates. The quality and quantity of RNA was determined using a NanoPhotometer spectrophotometer and gel electrophoresis. Eligible samples were submitted to BMK for Illumina HISEQ2000 high-throughput sequencing and paired-end reads were generated. Blackberry genome is used as a reference genome (https://www.rosaceae.org/species/rubus_occidentalis/genome_v1.1). Clean reads without adaptor sequences, poly-N containing or low-quality sequences were got after processing the raw reads. Meanwhile, Q20, Q30, GC-content of the clean reads were calculated. Sequencing Reads were aligned to the reference genome using the software TopHat2. Based on the results of TopHat2 alignment between Reads and reference sequences, the transcripts were spliced and quantified by Cufflinks.

To validate the high-throughput sequencing results, four target genes involved in secondary metabolite pathways of red raspberry were tested by qRT-PCR. Three biological replicates per sample were performed (Table 1). The cDNA was got using the Aidelai II RT Kit according to the manufacturer’s instructions, and qRT-PCR was carried out using the SYBR qPCR Master Mix Kit. The real-time PCR system with a 96-well plate was used to conduct the reaction. The conditions for the PCR amplifications were as follows: 95°C for 2 min, 95°C for 15 s, followed by 40 cycles of 55°C for 20 s, and 72°C for 25 s. A gene encoding actin was used as a reference. Relative expression levels of genes were calculated with the 2^-ΔΔCt^ method [13].

**Table 1:** Primer sequence information verified by qRT-PCR.

Gene	Primer sequence (5’ to 3’)
T27452	ATGTCTAGCCGAAGGTCAAGA
	GTCTACTATTGCGAATCTCAGGAAG
T10116	ACCGAGCGATGACCTTGT
	GTTGCGTTCCACCGTTATGT
T08677	GGAACAACAACAACAACCACAAC
	GGAACCAAGGACCGCAATG
T08811	ATGCTGTTGAGTATGTGAAGTTCTT
	CCGATGCCTATTCCATTGTTGA
Actin	TGCCATCCTTCGTCTTG
	CATCTGAAACGCTCTGC

### 2.3 Transcriptome bioinformatics analysis of red raspberry fruit

All the downstream analyses were based on clean data with high quality. Differential expression gene sequences were aligned by Blast X to the databases including NCBI non-redundant protein sequences (NR), NCBI non-redundant nucleotide sequence (NT), Kyoto Encyclopedia of Genes and Genomes (KEGG), Protein family (Pfam), Clusters of orthologous groups of proteins (KOG), A manually annotated and reviewed protein sequence database (Swiss-Port), KEGG orthology database (KO). According to the KEGG clustering results, the selected genes of phenylpropane metabolism pathway were compared by Blast on NCBI.

### 2.4 Analysis of differentially expressed genes

Differences in gene screening standards are very important. DEGseq software was used for screening differential genes, we used the standard |log_2_ (fold change)| ≥ 1 and padj ≤ 0.05 as common values. In order to identify possible key genes related to raspberry ketone, we used |log2 (fold change)| ≥ 5 as a selection criterion. Some genes with high expression levels were screened out from the list of differentially expressed genes (DEGs).

The biosynthesis of raspberry ketone begins with the synthesis of phenylalanine through the shikimic acid pathway, followed by the processes of phenylalanine deamination, methylation and hydroxyl activation, followed by further decarboxylation and condensation by the critical BAS to produce phenylmethylene acetone and finally raspberry ketone by the action of benzalacetone reductase. *BAS* and *CHS* belong to the plant type III polyketide synthase (PKS) superfamily. By BLAST comparison, a novel gene Bras_T16736, named *RinPKS4*, was obtained by further screening of PKS superfamily genes.

### 2.5 Production of transgenic raspberry over-expressing RinPKS4

Total RNA was isolated from red raspberry using the Plant Total RNA Isolation Kit (Autolabtech, Beijing, China). Reverse transcription was conducted at 42°C using the ImPromII^™^ Reverse Transcription System. The PCR program were as follows: 95°C for 5 min, followed by 30 cycles of 94°C for 30 s, 59°C for 40 s, and 72°C for 90 s, 72°C for 5 min. The amplified DNA was digested with *Sam* I/*Xba* I, and cloned into the p3301-121 vector. The constructed plasmid was transformed into *Agrobacterium tumefaciens* strains EHA105 and infected the leaves of red raspberry by injection. The transgenic plants were screened in the medium containing 1.5 mg/L glufosinate ammonium resistance (Table 2).

**Table 2:** Fluorescence quantitation primer sequences.

Gene	Primer sequence
RinPKS4-F	GCTCTAGAATGTCGAAAATACAAAGC
RinPKS4-R	TCCCCCGGGTTACAGGCTGCGAAGTAG
RinPKS4-F1	ACAGCCACCATCAGACAA
RinPKS4-R1	CACTTGAGGAGACATAGACAATG

Note: the underline represents the enzyme digestion base.

### 2.6 Determination of raspberry ketone content by HPLC and HPLC-MS

The leaves of the “Polka” and “Orange legend” were ground with liquid nitrogen, extracted with chloroform for 48 hours, and then steamed at 55°C to a powder, which was dissolved in 1 mL methanol, pass 0.22 μm organic filter membrane. Analysis of the products was performed by high-performance liquid chromatography (HPLC) on a Kromosil C18 reverse-phase column (5 μm, 250 mm×4.6 mm; Macherey-Nagel, Duren, Germany). The eluents were water (A) and methanol (B) at a flow rate of 1.0 ml min^-1^. The following gradients were used: 0%-30% B for 10 min, 30% B for 20 min, 30%-0% B for 5 min. The detection wavelengths were 275 nm.

For the on-line HPLC-MS analysis, liquid chromatography was performed on an Agilent 1290 infinity II HPLC system, which was coupled to an Agilent LCMS-6470 triple-quadrupole mass spectrometer with an electrospray ionization (ESI) interface (Agilent), and data acquisition and processing were performed using MassHunter WorkstationB 08 (Agilent). The MS conditions were as follows: drying gas temperature, 300°C; drying gas flow, 5 L min^-1^; nebulizer pressure, 45 psi; sheath gas temperature, 250°C; sheath gas flow, 11 L min^-1^; capillary voltage, 3500 V and nozzle voltage, 500 V.

## 3. Results

### 3.1 cDNA library construction and transcriptome assembly

In order to explore the difference of raspberry ketone content between the two red raspberry varieties, the transcriptomes of “Polka” and “Orange legend” at different stages were sequenced. Each sample sets three replicates. A total of 86.95 Gb clean data were obtained. After a rigorous quality assessment, Q20 in each sample is greater than 97.82%, Q30 is greater than 94%, with GC percentage ranging from 46%-48%. The results indicate that all the subsequent analyses based on the clean reads (Table 3). With blackberry as the reference genome, the proportion of the sequence matching to the reference genome database is about 81.47%-86.16%. This higher proportion of gene assembly results provides sufficient data for subsequent analysis of biological information.

**Table 3:** Summary of sample sequencing output statistics.

Sample	Clean Reads	Clean Bases	Error (%)	Q20 (%)	Q30 (%)	GC (%)	Mapped Reads (%)	Uniq Mapped Reads (%)
BER-qing1	23050400	6888446514	0.03	98.04	94.52	47.25	83.66	80.78
BER-qing2	25036022	7490194970	0.03	97.82	94.17	46.71	83.21	81.14
BER-qing3	20513099	6131186092	0.03	97.78	94.02	46.42	82.94	80.95
BER-hong1	20118313	6017549472	0.03	98.18	94.70	46.66	83.54	81.68
BER-hong2	20376947	6089086846	0.03	98.10	94.60	46.72	82.64	80.78
BER-hong3	23781885	7106059090	0.03	98.01	94.46	46.87	81.47	79.79
CSCQ-qing1	26010817	7733933104	0.03	98.47	95.38	46.56	85.25	81.13
CSCQ-qing2	23989790	7155352136	0.03	98.64	95.72	46.97	86.16	81.92
CSCQ-qing3	23182701	6899488816	0.03	98.19	94.73	46.89	84.62	80.33
CSCQ-hong1	311233349	9310919794	0.03	98.40	95.17	47.47	82.97	78.61
CSCQ-hong2	28031504	8349537928	0.03	98.56	95.52	47.24	82.78	78.56
CSCQ-hong3	26193024	7778158996	0.03	98.39	95.27	47.74	83.25	79.07

**Fig 2:**
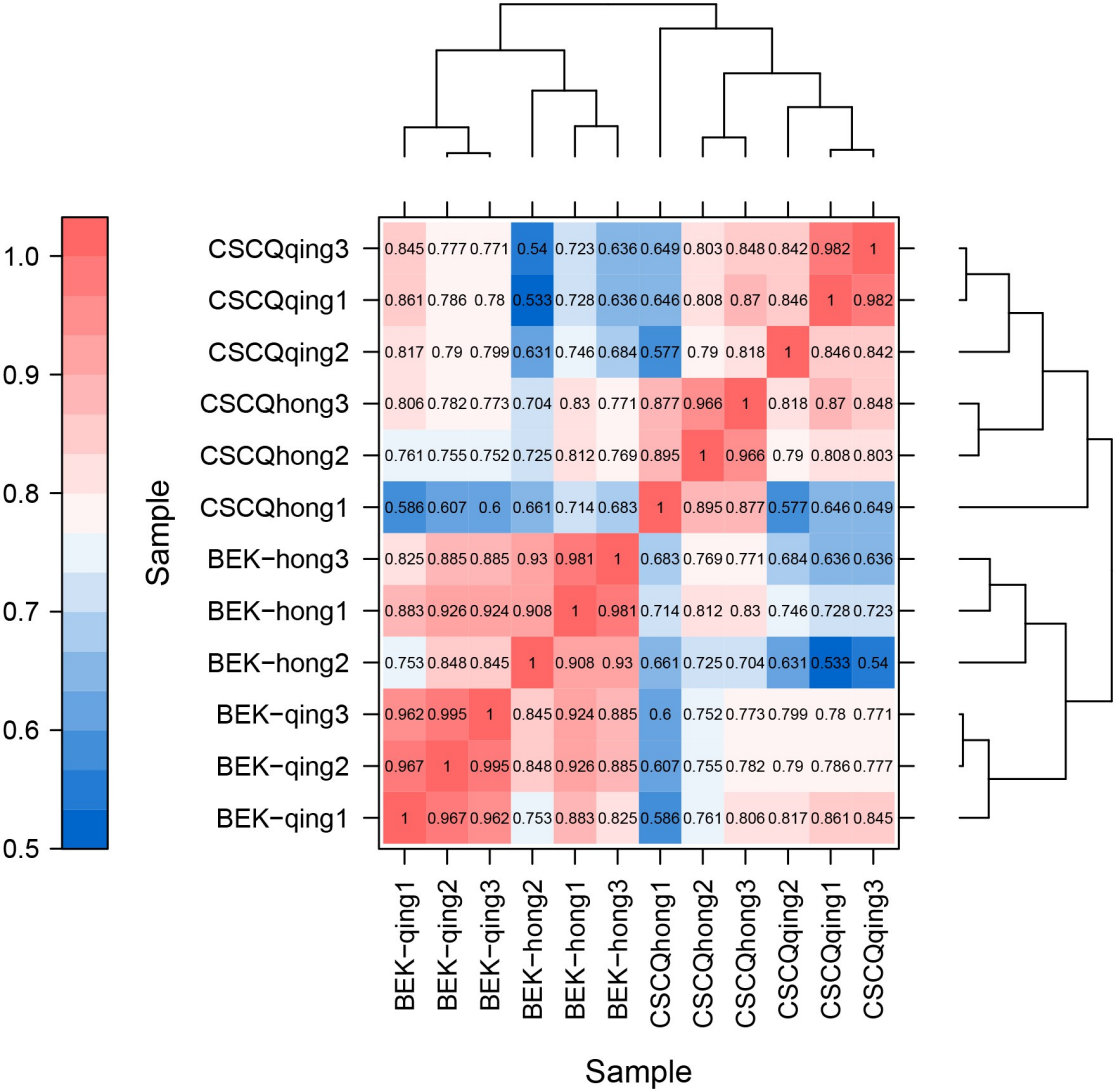
Correlation heat map; sample differences between groups and the sample repetition within groups. The left and upper sides are sample clustering, the right and lower sides are sample names, and the squares of different colors represent the correlations of the two samples.

In accordance with the FPKM values of all the genes in each sample, the correlation coefficients of samples within and between groups were calculated, and a heat map was constructed that can visually display the sample differences between groups and the sample repetition within groups. The higher the correlation coefficient between samples, the closer the expression pattern. The correlation coefficient of the three biological replicates for each growth and development period was close to 1, which showed that the data of the three biological replicates at each stage were very good, and the correlations within and between groups were good. Thus, they can be used for subsequent experiments (Fig 2).

**Fig 3:**
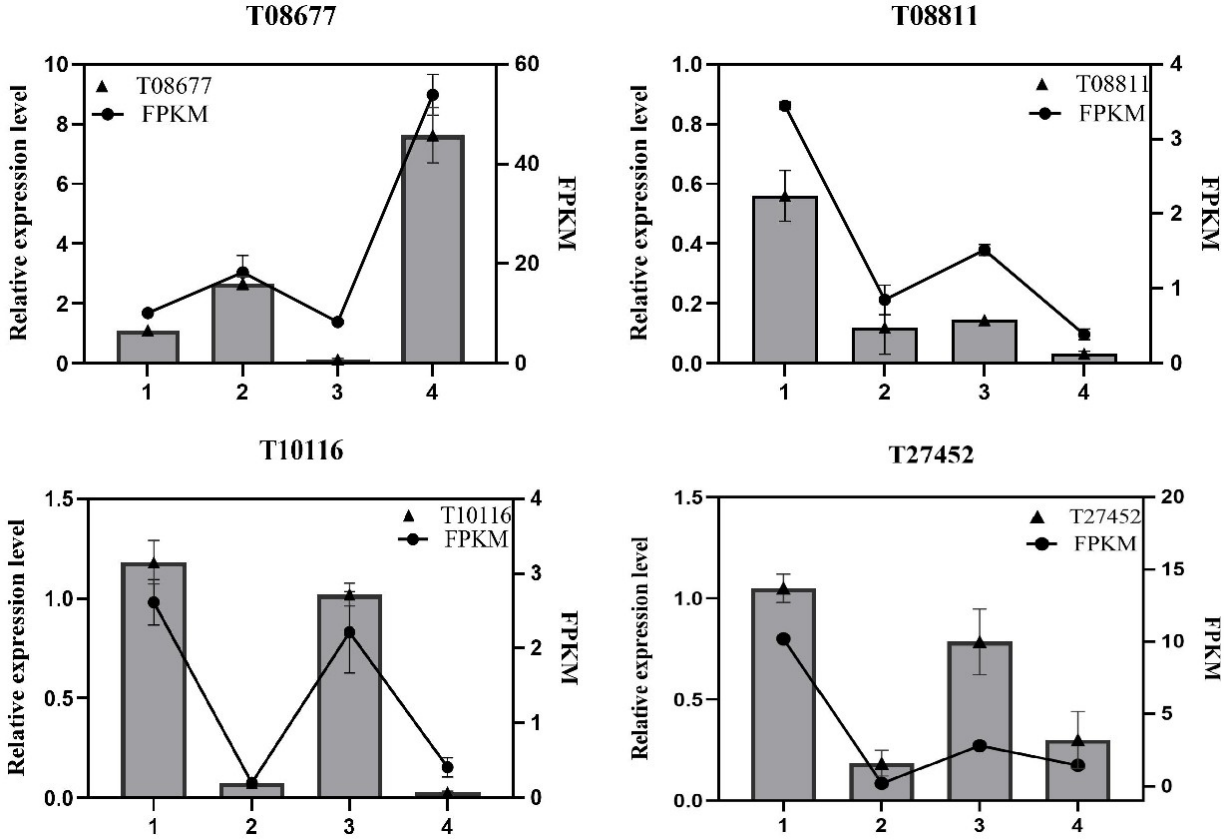
Quantitative real-time polymerase chain reaction analysis of the expression of secondary metabolism-related genes (Analyses were completed in triplicate) 1: The green fruit from Polka; 2: The ripe fruit from Polka; 3: The green fruit from Orange legend; 4: The ripe fruit from Orange legend.

The qRT-PCR experiment was performed to confirm the reliability of the RNA-seq data using the actin as an internal control and four genes as verification targets. The expression patterns of four genes are well consistent with the results of RNA sequences, suggesting that the RNA sequencing data are reliable (Fig 3).

### 3.2 Annotation and classification of gene functions

In order to get a comprehensive annotation of gene function, 24213 unigenes were annotated using several databases (Table 4). In the seven databases, the number of unigenes are highly similar to entries in the NR (99.48%), NOG (91.86%), COG (35.21%), SwissProt (73.21%), Pfam (78.22%), GO (58.83%), KEGG (37.88%) or KOG (53.68%). Based on the Blast results, 2,030 new genes were found, of which 1,731 had functional annotation and 299 had no annotation information.

**Table 4:** Success rate of genes annotated in seven databases.

Databases	Number of Genes	Percentage(%)
Annotated in NR	24088	99.48
Annotated in NOG	22242	91.86
Annotated in COG	8526	35.21
Annotated in SwissProt	17727	73.21
Annotated in Pfam	18941	78.22
Annotated in GO	14246	58.83
Annotated in KEGG	9171	37.88
Annotated in KOG	12997	53.68
Total Unigenes	24213	100

**Fig 4:**
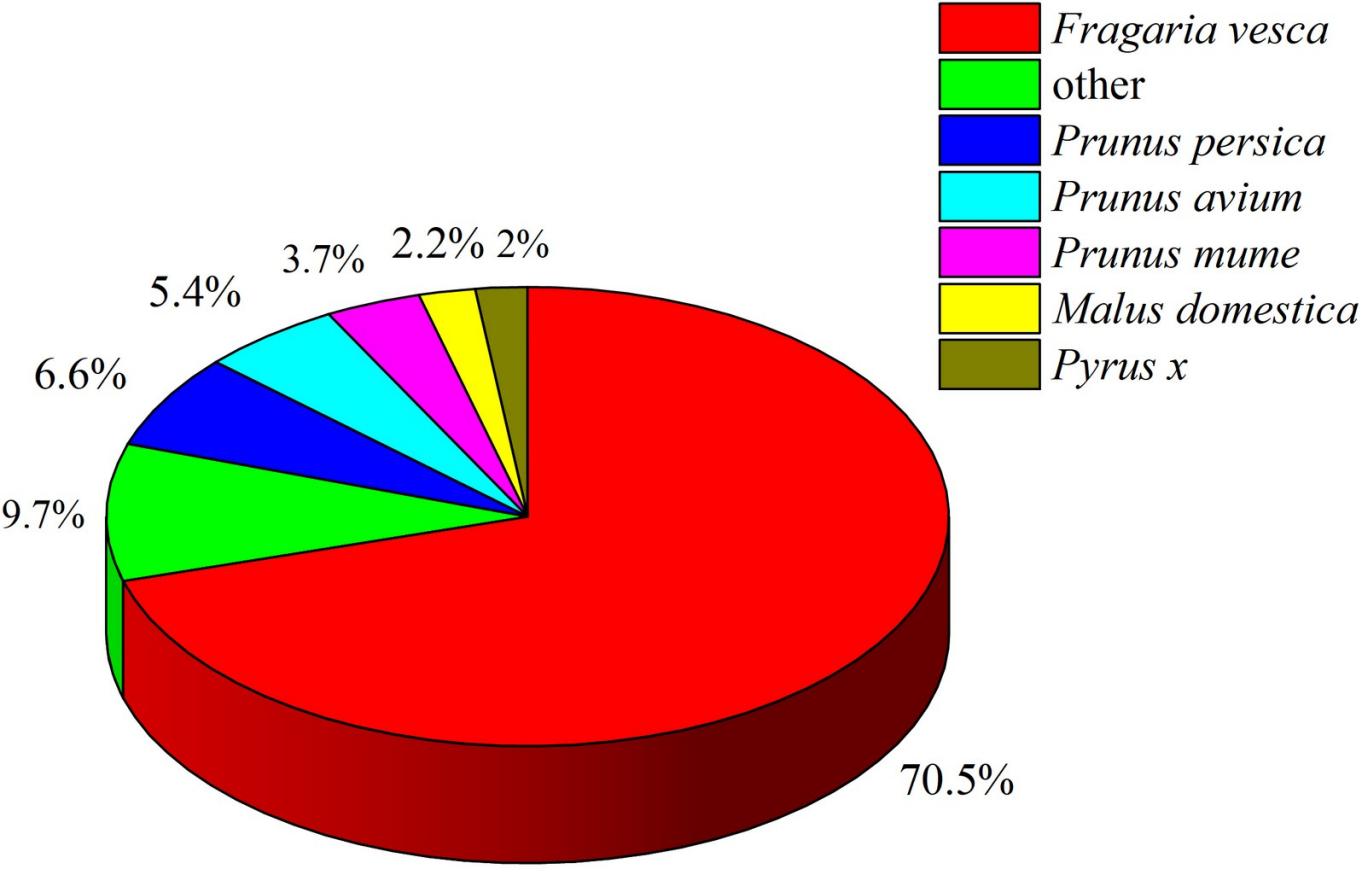
Comparison of NR protein database annotated genes with other species.

All unigenes were aligned against the NR protein databases of GenBank using BLAST. The results indicate that a majority of best hits (70.5%) are *Fragaria vesca*. Moreover, 6.6% unigenes is similarity with *Prunus persica* followed by 5.4% with *Prunus avium*. It is noteworthy that about 9.7% unigenes are identified with other species (Fig 4).

**Fig 5:**
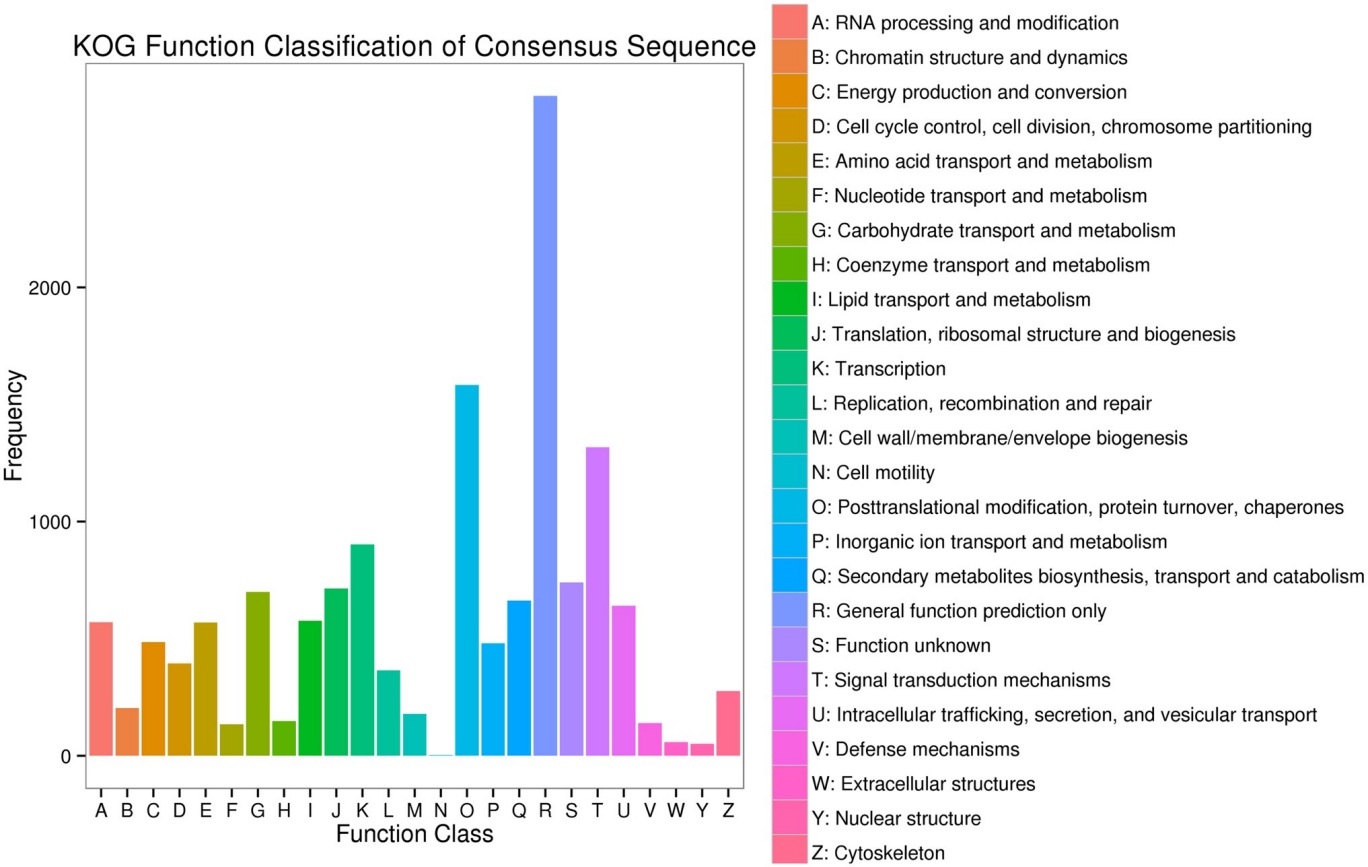
Functional annotation and classification based on KOG database.

By blasting against the KOG database to predict and classify function of gene, the 24213 unigenes were assigned to 25 categories, including the categories of general function prediction only (2817), posttranslational modification, protein turnover and chaperones (1583), signal transduction mechanisms (1317), Extracellular structures (58), Cytoskeleton (51), and other categories (Fig 5).

**Fig 6:**
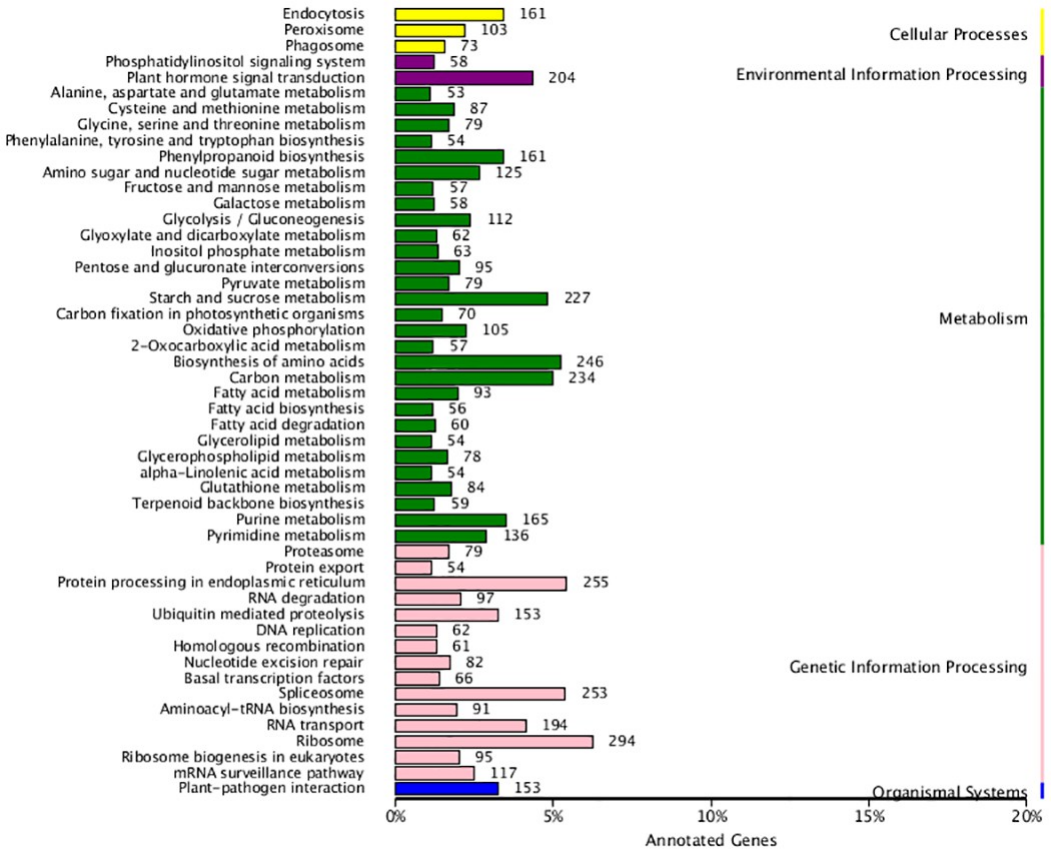
Functional annotation and classification based on KEGG database.

KEGG analysis system was used to assigned the unigene metabolic pathway. The 9171 unigenes were divided into five primary KEGG categories (Fig 6), namely, Cellular Process, Environmental Information Processing, Metabolism, Genetic Information Processing, and Organismal Systems. The Cellular Process category includes 3 secondary categories, Environmental Information Processing includes 2 secondary categories, Metabolism includes 29 secondary categories, the most genes related to Biosynthesis of amino acids were 246, followed by Carbon metabolism, Starch and sucrose metabolism. Alanine, aspartate and glutamate metabolism were the fewest, with only 53 genes. It is worth to watch the number of Phenylpropanoid biosynthesis genes were 161. Genetic Information Processing includes 15 secondary categories, the number of Ribosome genes was 294, and Organismal Systems includes 1 secondary category.

### 3.3 Screening for differential expressed genes

**Fig 7:**
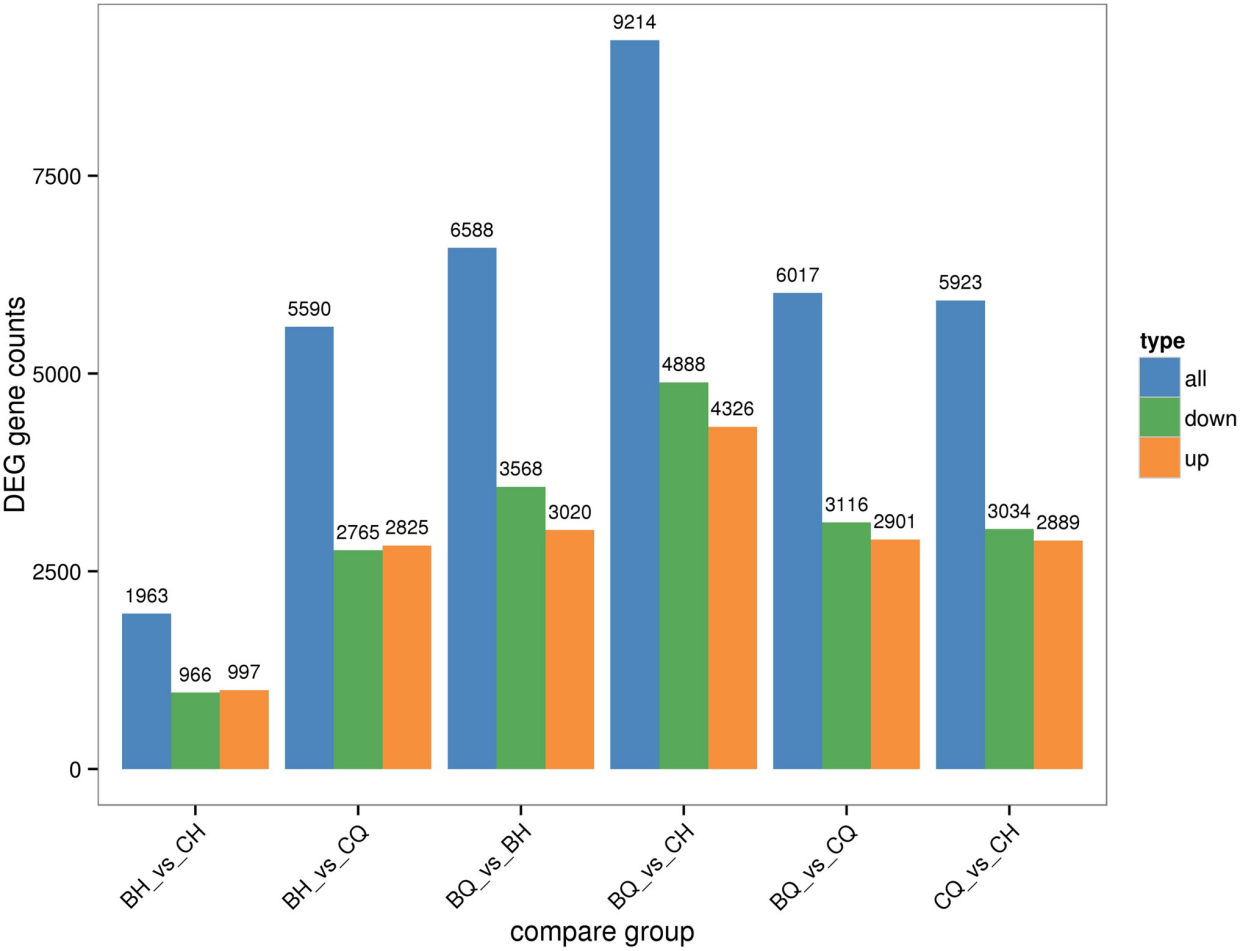
Differentially expressed genes of red raspberry in different comparison groups; BQ: The green fruit from Polka; BH: The ripe fruit from Polka; CQ: The green fruit from Orange legend; CH: The ripe fruit from Orange legend.

In different stage, there were more down-regulated genes than up-regulated genes, indicating that the ripe stage on the gene expression trend was mainly down-regulated (Fig 7). To further analyze the differences in gene expression functions in different stage, DEGs in each group were subjected to a KEGG pathway enrichment analysis. From the KEGG enrichment results, the 20 most significant KEGG pathways were selected, and scatter diagrams were constructed. If there were less than 20 pathways, all the pathways were included. In the BC vs CC, the largest number of differential genes annotated to the pathway was Carbon metabolism, and the number of DEGs was 31. In the BC vs CQ, the largest number of DEGs was annotated to the pathway of Carbon metabolism, having 91 DEGs. In the BQ vs BC, the largest number of DEGs was annotated to the pathway of Ribosome, having 124 DEGs. The second most annotated pathway was Biosynthesis of amino acid, with 99 DEGs. In the BQ vs CC, the largest number of DEGs was annotated to the pathway of Ribosome, having 183 DEGs. And in the BQ vs CQ and CQ vs CC, the DEGs number in Ribosome is largest (S2 Fig).

### 3.4 Analysis of differentially expressed genes

The key signaling pathways that affect raspberry ketone belong to phenylpropanoid biosynthesis pathway in metabolism. After Blast comparison, the most comments are peroxidase. The Blast of amino acid sequence did not directly annotate or predict the benzalacetone synthase (BAS) family genes (Table 5). BAS is a key enzyme in the ketone synthesis pathway of red raspberry, and CHS is a competitive enzyme in the ketone synthesis pathway of red raspberry. Both of them belong to the plant type III polyketide synthase (PKS) superfamily. By BLAST comparison, a novel gene Bras_T16736, named *RinPKS4*, was obtained by further screening of PKS superfamily genes.

**Table 5:** Analysis of known genes of phenylpropane metabolism pathway in red raspberry transcriptome.

Enzyme family	Number	Enzyme family	Number
phenylalanine ammonia lyase	2	anthocyanidin 3-O-glucosyltransferase	2
4-coumarate-Coenzyme A ligase	8	aldehyde dehydrogenase family	2
Cinnamate-4-hydroxylase	1	flavonoid 3’-O-methyltransferase	4
cinnamyl alcohol dehydrogenase	9	acylsugar acyltransferases	2
caffeoyl shikimate esterase	3	vicianin hydrolase-like	2
peroxidase	54	peroxiredoxin	1
beta-glucosidase	32	Shikimate O-hydroxycinnamoyl transferase	1
caffeic acid-O-methyltransferase	8	cytochrome P450	7
mannitol dehydrogenase	11	DNA topoisomerase	1
Spermidine hydroxycinnamoyl transferase	5		

### 3.5 Over-expression of RinPKS4 gene in red raspberry

RNA was extracted from “Polka” and “Orange legend” leaves and reverse transcribed into cDNA. RinPKS4-F and RinPKS4-R were used as primers to clone *RinPKS4* gene. The gene was ligated with the p-TOPO cloning vector and transferred into *E*. *coli Trelief*^*™*^ *5α*. The *E*. *coli* was spread on the Ampicillin screening medium. The full-length *RinPKS4* gene is 1164 bp, which was the same as that of the result of transcriptome analysis (S3 Fig).

The expressing vector p3301-121 was digested with *Sma* I and *Xba* I, and the linear *RinPKS4* cDNA fragment was recovered and ligated with the linear fragment of the gene. The vector was transformed into *Trelief*^*TM*^ *5ɑ* and spread on the screening medium containing kanamycin, single colonies were selected and were sequenced. The recombinant strain p3301-121-*RinPKS4* was sequenced, purified and verified by restriction endonuclease digestion with *Sma* I and *Xba* I. After electrophoresis detection, the plant over-expression vector p3301-121-*RinPKS4* was constructed successfully (S3 Fig).

The plant over-expression vector p3301-121-*RinPKS4* was transformed into *agrobacterium tumefaciens* and coated on the screening medium containing kanamycin and rifampicin resistance. Using RinPKS4-F1 and RinPKS4-R1 primers, the colonies containing the recombinant plasmid were verified by PCR and sequenced. The results of sequencing were consistent with the inserted target fragment, and the *agrobacterium*-mediated transformation of EHA105 was successful.

**Fig 8:**
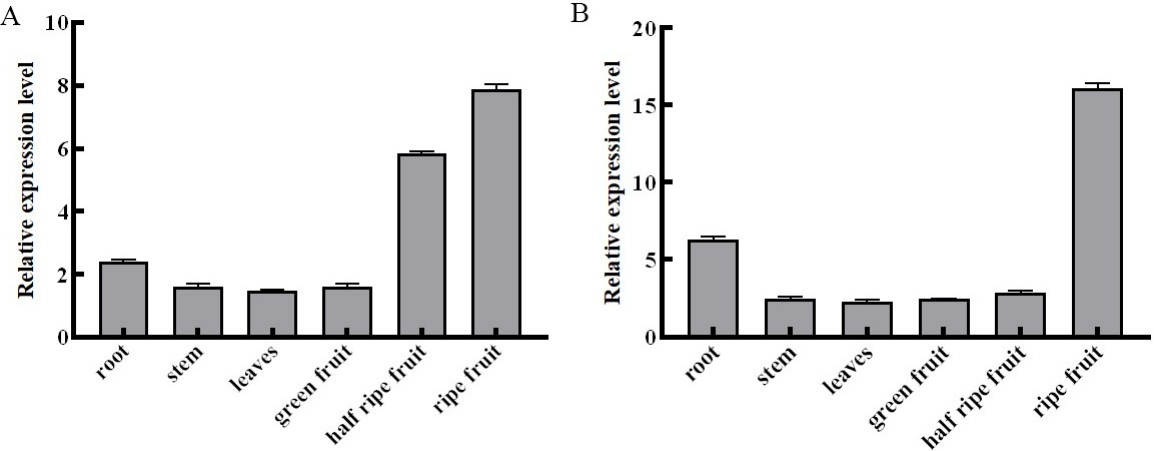
Over-expression of *RinPKS4* gene in red raspberry. A: *RinPKS4* expression analysis of each tissue part of “Polka”; B: *RinPKS4* expression analysis of each tissue part of “Orange legend”.

The expression levels of *RinPKS4* gene in different tissues and development stages (green fruit, half ripe fruit, ripe fruit) of red raspberry were detected. The results showed that the highest expression was found in ripe fruits, followed by half ripe fruits and the lowest in green fruits, which was contrary to the trend of raspberry ketone content during development stage. The expression level of *RinPKS4* in the roots, stems and leaves of the two red raspberry varieties was relatively high, while that in the fruit of “Polka” was the lowest (Fig 8A), the gene expression levels in root, stem, leaf, green fruit and half ripe fruit were relatively high in “Orange legend” (Fig 8B).

### 3.6 Detection of the content of raspberry ketone in over-expressing RinPKS4 transgenic red raspberry

**Fig 9:**
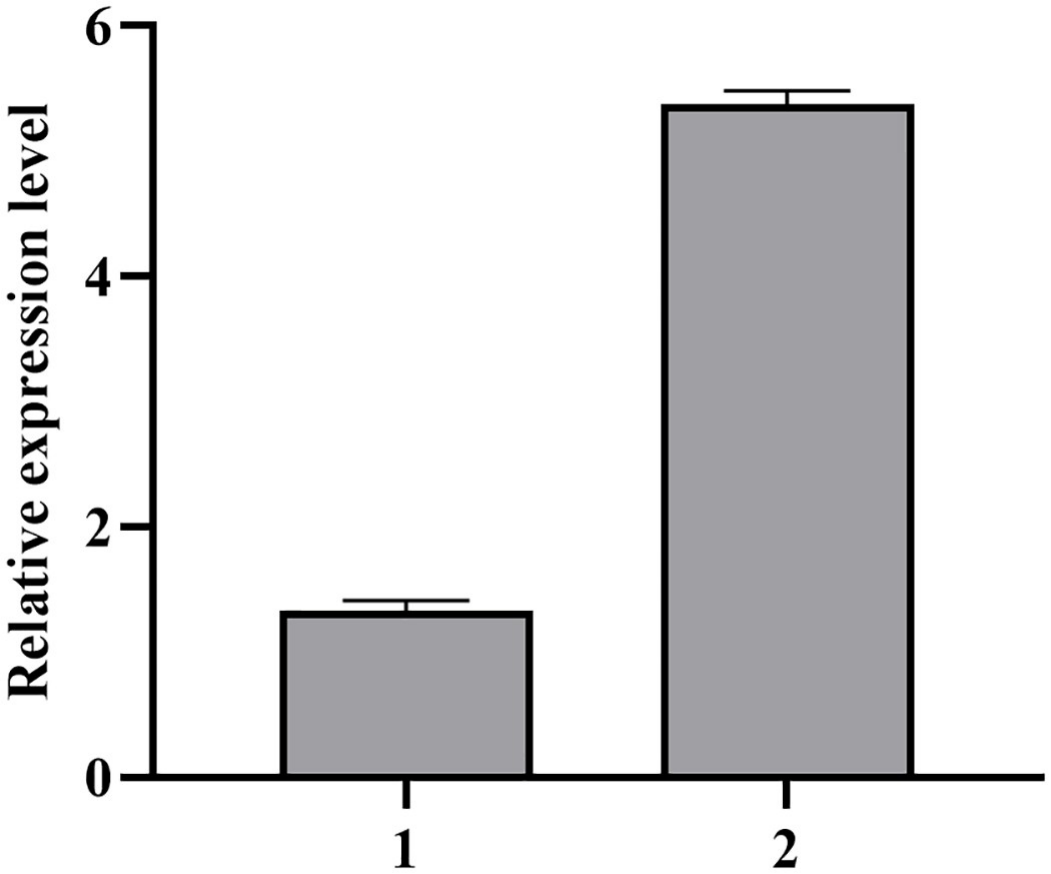
Relative expression level of *RinPKS4* in transgenic red raspberry. Bar 1 is the expression of *RinPKS4* gene in p3301-121 transgenic strain; bar 2 is the expression of *RinPKS4* gene in p3301-121-*RinPKS4* transgenic lines.

RNA was extracted from p3301-121 and p3301-121-*RinPKS4* transgenic lines (S4 Fig). The RNA was reverse transcribed into cDNA and the *RinPKS4* gene was detected by qRT-PCR. The results of qRT-PCR showed that the expression level of *RinPKS4* was 1.273±0.177 in p3301-121 transgenic lines. After over-expressing *RinPKS4*, the expression level of *RinPKS4* was 5.29±0.194, gene expression was significantly increased (Fig 9).

The 0.05 mg standard raspberry ketone was dissolved in 1 ml chromatographic grade methanol and passed through 0.22 μm organic filter membrane to detect the standard raspberry ketone. The results of HPLC showed that the target peak appeared at 16 min. The content of raspberry ketone in p3301-121 transgenic line was 2.65 μg/g, and that in p3301-121-*RinPKS4* transgenic line was 3.78 μg/g. The raspberry ketone content of p3301-121-*RinPKS4* transgenic line was 42.64% higher than that of p3301-121 transgenic line, and the increase of raspberry ketone content was more obvious (S5 Fig).

## 4. Discussion

Red raspberry has a unique aroma, which is different from other fruits, directly affects the flavor quality of red raspberry [14]. It was found that the unique aroma was derived from raspberry ketone, a characteristic effect compound in red raspberry [15].

The synthetic pathway of raspberry ketone has been proved to be derived from the phenylpropane metabolic pathway. In this pathway, Benzalacetone synthase (BAS) catalyzes the 4-coumaroyl-CoA with malonyl-CoA to form phenylmethylacetone, a precursor of raspberry ketone. It is concluded that BAS is the key enzyme to control the formation of raspberry ketone. However, it is difficult to isolate the members of the plant type III polyketide synthase (PKS) superfamily by homologous cloning. In recent years, although a lot of research work has been done in red raspberry, but *BAS* gene never has been isolated from red raspberry [16]. On this basis, we used Illumina HiSeq high-throughput sequencing platform, based on raspberry ketone content changes at different development stages, the transcriptomes of “Polka” and “Orange legend” were sequenced at different stages, and 86.95 GB of Clean Data were obtained. After assembly, 24213 single genes were got.

**Fig 10:**
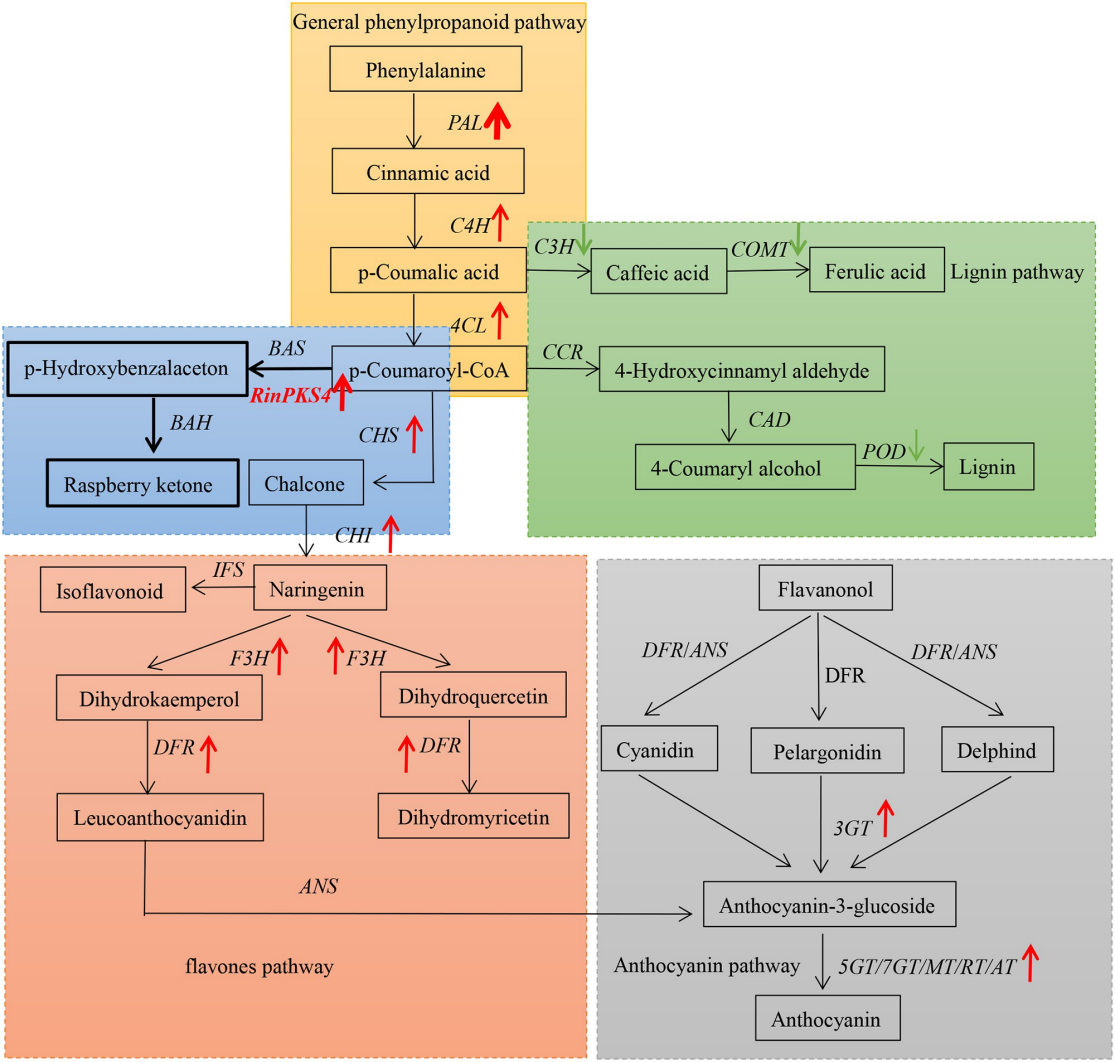
Schematic diagram of phenylpropanoid pathway; red and green arrows indicate the up regulation of gene expression and the down regulation of gene expression and the width of the arrows as a more visual representation of the fold change in gene expression. A: General phenylpropanoid pathway; B: Lignin pathway; C: Anthocyanin pathway; D: flavones pathway.

Based on Illumina sequencing technology, the large-scale transcriptome sequencing data in red raspberry were generated. The unigenes were also analyzed against NR, GO, KOG and KEGG. Among the annotated sequences, the species with the highest number of best hits were *Fragaria vesca*, the similar result was found for blackberry [17] and Korean raspberry [18]. These results were consistent since strawberry is the species closest to *Rubus sp*. with sequenced genomes, all belonging to the family Rosaceae, which also reflected the scientific nature of botany classification from the side [19]. In the KEGG database, 9171 single genes were classified into five major categories, with the largest number focusing on metabolic engineering, reflecting the rich metabolic activities and complex regulatory processes of mangrove. In addition, 299 genes did not receive functional annotation information, either because they contained non-coding sequences or because they were unique to the red raspberry [20]. Phenylpropanoid pathway includes lignin pathway, anthocyanin pathway and flavones pathway. In the process from green fruit to ripe fruit, the genes involved in lignin synthesis are significantly downregulated, which leads to the metabolism flow to the synthesis of flavonoids, and the key genes involved in the synthesis of flavonoids are upregulated (Fig 10). The same substrate was used for raspberry ketone and chalcone, and CHS gene expression increased during ripe stage. As a result, the expression level of raspberry ketone at the green fruit stage was significantly higher than that at the ripe fruit stage.

In this experiment, the content of raspberry ketone in different stage was detected, and the two red raspberry varieties were screened for transcriptome sequencing. *RinPKS4* was obtained by bioinformatics analysis of transcriptome data, which is highly related to the change of raspberry ketone content. It was found that *RinPKS4* plays an important role in the synthesis of raspberry ketone by analyzing the relationship between tissue-specific expression and the content of raspberry ketone. In particular, the over-expression of *RinPKS4* was positively correlated with the change of raspberry ketone content, indicating that *RinPKS4* gene had a direct regulatory effect on the production of raspberry ketone. In addition, we also found the change rule of the content of raspberry ketone and the expression level of key genes in the synthesis of raspberry ketone at different development stages. It laid a foundation for isolating the key *BAS* gene in red raspberry and improving the quality of red raspberry fruit. On the one hand, transcriptome data provide the data base for red raspberries. It can be used by other researchers to learn more about red raspberries. On the other hand, the discovery of *RinPKS4* gene also provides a theoretical basis for the subsequent research to increase the content of raspberry ketone by molecular breeding methods. So more raspberry ketone can be produced through the method of biologically.

The method is used to produce red raspberries with high raspberry ketone content and improve the quality of red raspberries.

## Supporting information

S1 Fig The biosynthetic pathway of raspberry ketone. (TIF)

S2 Fig Volcano diagram of differential gene expression levels in each comparison group. (A) BC vs. CC; (B) BC vs. CQ; (C) BQ vs. BC; (D) BQ vs. CC; (E) BQ vs. CQ; (F) CQ vs. CC; BQ: the green fruit from Polka; BH: the ripe fruit from Polka; CQ: the green fruit from Orange legend; CH: the ripe fruit from Orange legend.
(TIF)

S3 Fig Over-expression of *RinPKS4* gene in red raspberry. A: *RinPKS4* conserved sequence; lane M: 2000^+^Market; lane 1–3: the target gene band of *RinPKS4*; B: Construction of p3301-121-*RinPKS4* over-expression vector; lane M: 15000 Market, lane 1–2: p3301-121-*RinPKS4* digestion.
(TIF)

S4 Fig Red raspberries grow state, 1 is the growth of red raspberry without glufosinate; 2 is the growth of red raspberry with 1.5 mg/L glufosinate; 3 is the growth situation of over-expressing p3301-121 transgenic lines; 4 is the growth situation of over-expressing p-3301-121-*RinPKS4* transgenic lines. (TIF)

S5 Fig Detection of raspberry ketone content. 1 is the chromatogram of raspberry ketone standard; 2 is the chromatogram of raspberry ketone of transgenic p3301-121 line; 3 is the chromatogram of raspberry ketone of p3301-121-RinPKS4 transgenic line.
(TIF)

S6 Fig Phenylpropane metabolism map in KEGG. (TIF)

S7 Fig Raspberry ketone mass spectrum identification of resveratrol by HPLC-MS. A: The resveratrol peak (163.0) was shown in Full Scan LC-MS. B: The product ion peaks of resveratrol (123 and 91) were shown in the Multiple Reaction Monitoring (MRM) scan.
(TIF)
