# Supplementary figures and images for: A new raspberry ketone synthesis gene RinPKS4 identified in Rubus idaeus L. by transcriptome analysis
Source: PLoS One. 2024 Aug 22;19(8):e0306615. doi: 10.1371/journal.pone.0306615 (PMC11341048; doi:10.1371/journal.pone.0306615)

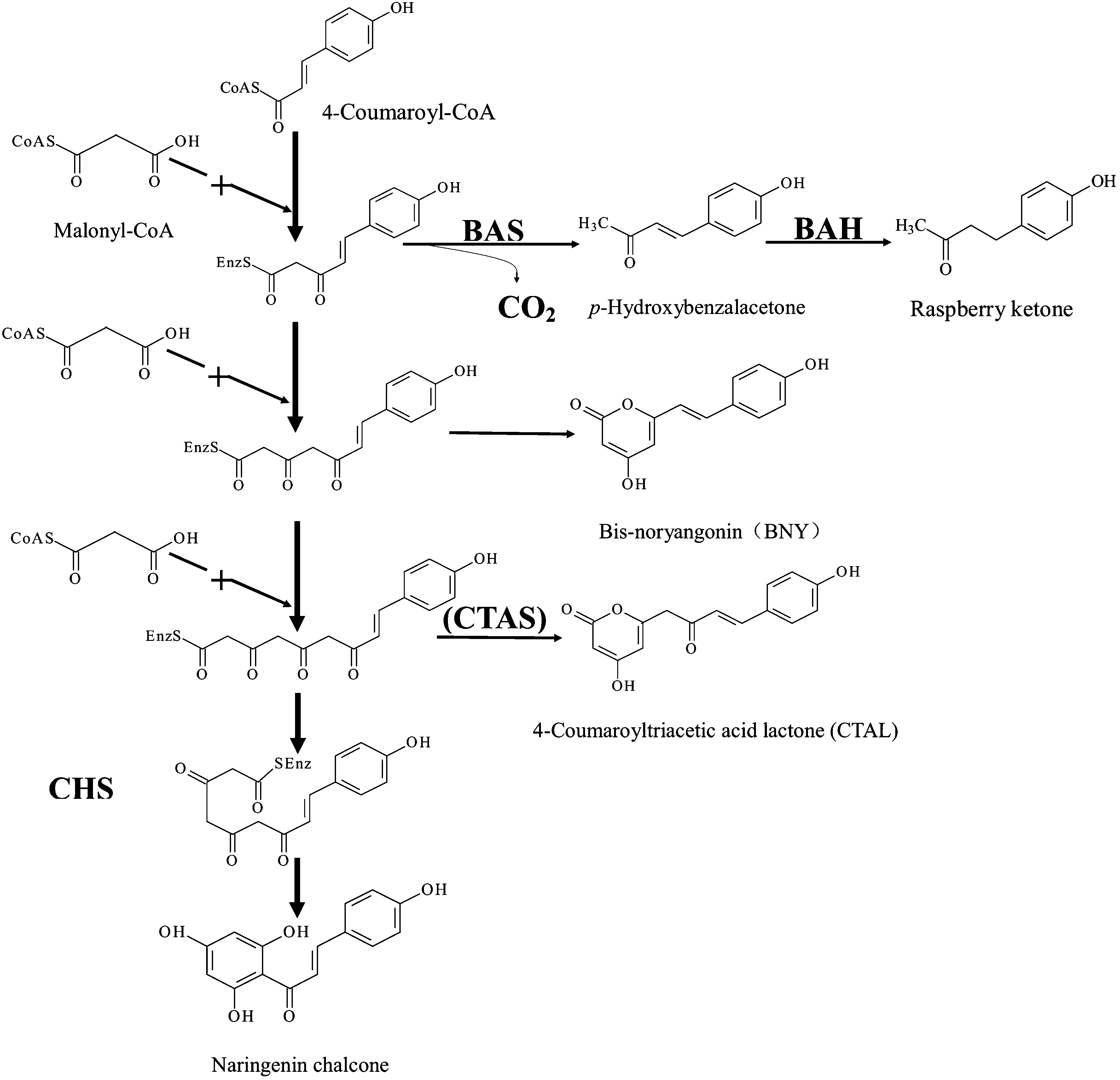

Supplement: S1 Fig — (TIF) [file pone.0306615.s001.tif]

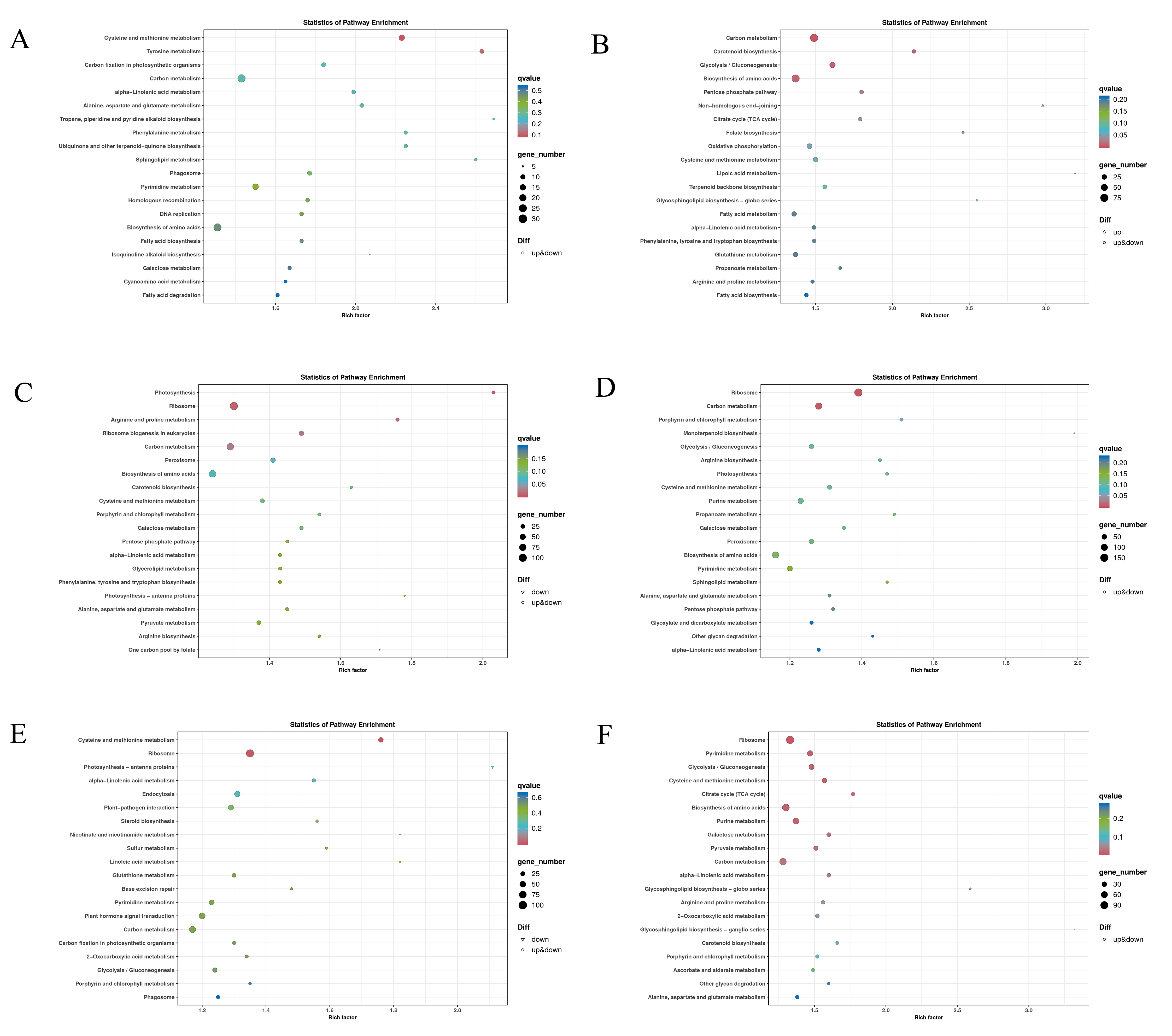

Supplement: S2 Fig — (A) BC vs. CC; (B) BC vs. CQ; (C) BQ vs. BC; (D) BQ vs. CC; (E) BQ vs. CQ; (F) CQ vs. CC; BQ: the green fruit from Polka; BH: the ripe fruit from Polka; CQ: the green fruit from Orange legend; CH: the ripe fruit from Orange legend. (TIF) [file pone.0306615.s002.tif]

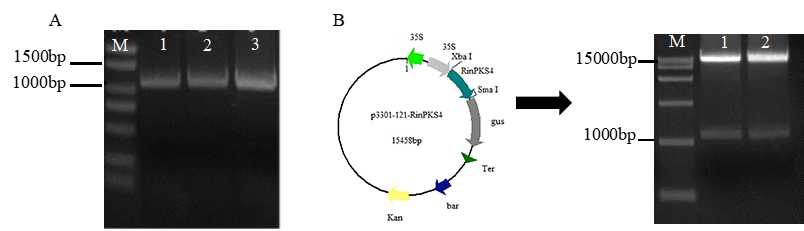

Supplement: S3 Fig — A: RinPKS4 conserved sequence; lane M: 2000+Market; lane 1–3: the target gene band of RinPKS4; B: Construction of p3301-121-RinPKS4 over-expression vector; lane M: 15000 Market, lane 1–2: p3301-121-RinPKS4 digestion. (TIF) [file pone.0306615.s003.tif]

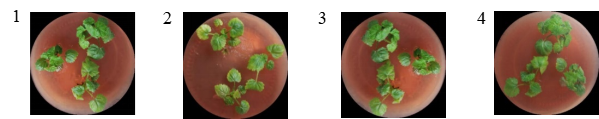

Supplement: S4 Fig — (TIF) [file pone.0306615.s004.tif]

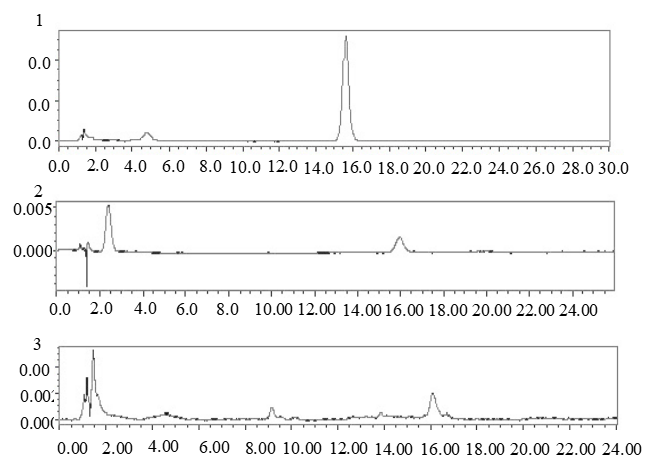

Supplement: S5 Fig — 1 is the chromatogram of raspberry ketone standard; 2 is the chromatogram of raspberry ketone of transgenic p3301-121 line; 3 is the chromatogram of raspberry ketone of p3301-121-RinPKS4 transgenic line. (TIF) [file pone.0306615.s005.tif]

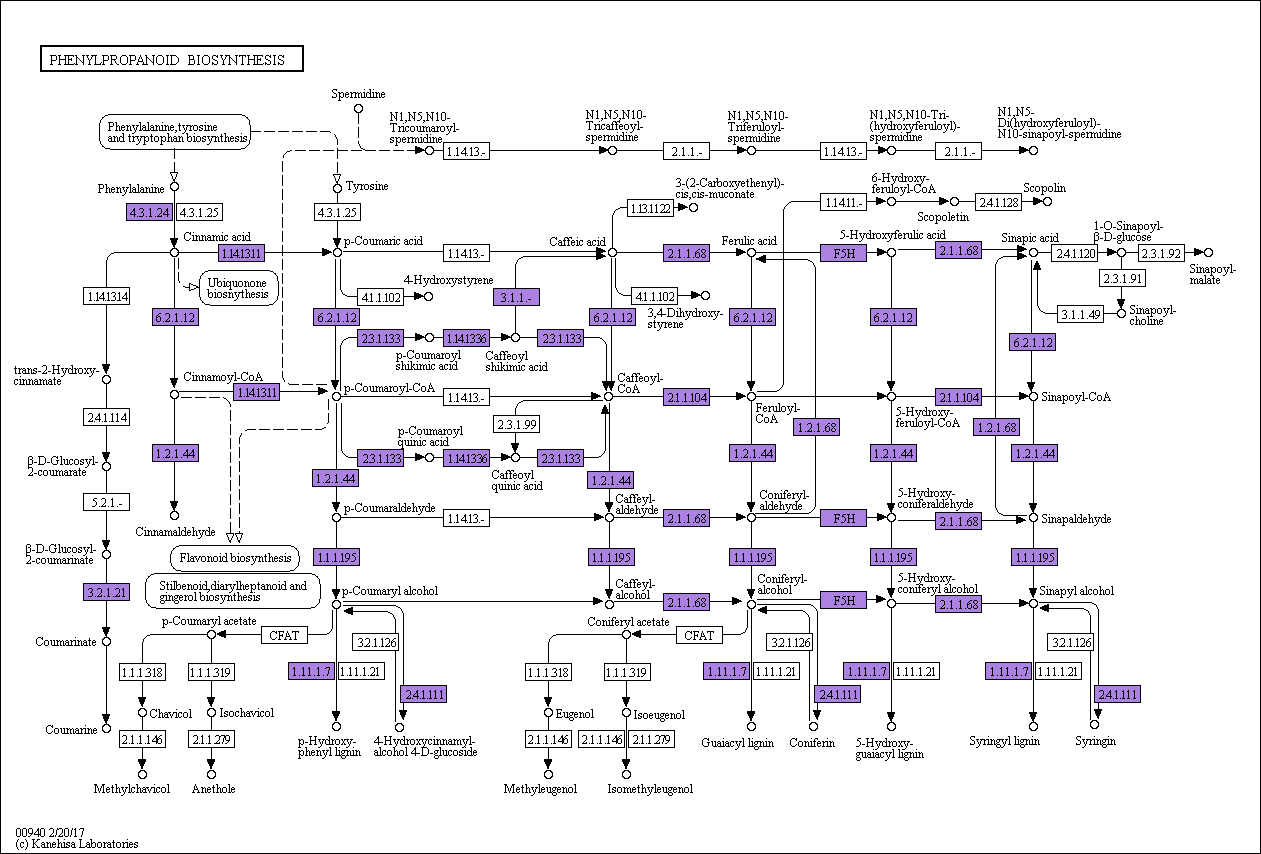

Supplement: S6 Fig — (TIF) [file pone.0306615.s006.tif]

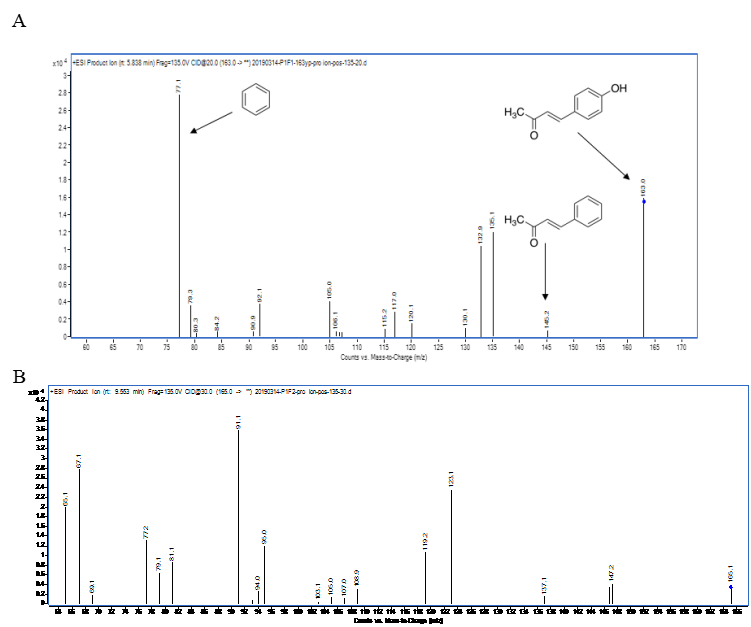

Supplement: S7 Fig — A: The resveratrol peak (163.0) was shown in Full Scan LC-MS. B: The product ion peaks of resveratrol (123 and 91) were shown in the Multiple Reaction Monitoring (MRM) scan. (TIF) [file pone.0306615.s007.tif]
